# How I faced my prostate cancer: a molecular biologist’s perspective

**DOI:** 10.1038/s41698-021-00229-5

**Published:** 2021-09-24

**Authors:** Monica Zuradelli, Massimo Lazzeri, Egesta Lopci, Paolo Andrea Zucali, Luca Balzarini, Giorgio Guazzoni, Piergiuseppe Colombo, Marta Scorsetti, Ciro Franzese, Rosanna Asselta, Giulia Soldà, Stefano Duga

**Affiliations:** 1 Department of Biomedical Sciences, Humanitas University, Via Rita Levi Montalcini 4, 20090 Pieve Emanuele Milan, Italy; 2 IRCCS Humanitas Research Hospital, Department of Oncology, Via Manzoni 56, 20089 Rozzano Milan, Italy; 3 IRCCS Humanitas Research Hospital, Department of Urology, Via Manzoni 56, 20089 Rozzano Milan, Italy; 4 IRCCS Humanitas Research Hospital, Nuclear Medicine, Via Manzoni 56, 20089 Rozzano Milan, Italy; 5 IRCCS Humanitas Research Hospital, Department of Radiology, via Manzoni 56, 20089 Rozzano Milan, Italy; 6 IRCCS Humanitas Research Hospital, Department of Pathology, Via Manzoni 56, 20089 Rozzano Milan, Italy; 7 IRCCS Humanitas Research Hospital, Department of Radiotherapy and Radiosurgery, Via Manzoni 56, 20089 Rozzano Milan, Italy; 8 IRCCS Humanitas Research Hospital, Via Manzoni 56, 20089 Rozzano Milan, Italy

**Keywords:** Molecular medicine, Cancer

## Abstract

Hippocrates (Kos, c.460–c.370 BC) reminds us that “It is more important to know what sort of person has a disease than to know what sort of disease a person has”. This is still true today and reflects the emerging role of personalized medicine for patient-specific risk stratification and treatment programs. This report documents my personal experience as a patient with aggressive prostate cancer, who, as a scientist, had the privilege to access cutting-edge medical care and molecular profiling.

The 6th of June 2016 would have been a wonderful Milanese spring day if only I (SD) had not received the result of my PSA test (26 ng/ml)—prescribed for recurrent lower urinary tract symptoms and inconclusive digital rectal examination—which suggested a possible prostate cancer (PCa). After a phlogistic cause was excluded, I underwent an mpMRI that evidenced a PI-RADS v2.0-V lesion in the right lobe extending to homolateral seminal vesicle, and subsequently to a 5-core target fusion biopsy combined with a 12-core systematic biopsy as per European Association of Urology guidelines. The pathology report confirmed, in all cores, a prostatic acinar adenocarcinoma, Gleason Score 5 + 5. A staging total body (11C)Choline-PET/CT demonstrated that the disease had already spread to regional iliac lymph nodes but with no distant visceral metastases noted.

After a multidisciplinary team discussion and considering the aggressiveness of the disease, its extension and the possible related local complications, I was submitted to an open radical retropubic prostatectomy with regional extended lymphadenectomy. The final pathology report confirmed a bilateral prostatic acinar adenocarcinoma, Gleason 5 + 5, with endocrine differentiation in <5% of cells (Synaptophysin+, CD56−, Chromogranin−). Vascular and perineural invasion was present. The disease involved 65% of the gland, with extra capsular invasion to both seminal vesicles. Seven of 22 dissected loco-regional lymph nodes resulted positive for disease. Final stage (TNMv8) was pT3bN1R1.

During the first urological visit in 2016, I was asked about the presence of male relatives who had developed PCa (none). At that time, the notion that a germline mutation in a DNA damage repair (DDR) gene could be related to a higher risk of aggressive PCa^1–3^ had not yet been implemented in common medical practice and I was not asked about the occurrence of breast/ovarian cancer in my family. However, my mother had died at 41 years due to aggressive breast cancer, therefore a suspicious familiarity for DDR gene-related cancer was present, even if no molecular information was available on a DDR gene mutation in my family.

Nonetheless, I was fortunate enough to lead a laboratory of molecular genetics that was just starting to work on PCa; thus I decided to perform whole-exome sequencing (WES) on my DNA. A first analysis focused on potentially pathogenic germline mutations in 20 DDR genes associated with autosomal-dominant cancer predisposition^3^. A total of 140 variants in 15 genes were detected (Supplementary Fig. 1A), of which 50 were exonic and 21 were missense variants. Only 10 of the 140 identified variants had a frequency ≤1% in the general population (GnomAD database). Among these, the best candidates were 2 missense variants, both with CADD score ≥20: NM_032043.2(*BRIP1*):c.790C>T (p.Arg264Trp) and NM_000059.3(*BRCA2*):c.8375T>C (p.Leu2792Pro) (Supplementary Fig. 1B).

The p.Arg264Trp variant in *BRIP1* (rs28997569) is annotated in Clinvar (Accession number VCV000128195) with conflicting interpretations, although most annotations report it as likely benign in the context of cancer predisposition^4^. In-silico predictions also gave discordant results, with 8 software annotating the variant as tolerated/neutral and 6 as deleterious/probably pathogenic (Supplementary Fig. 1C). The p.Leu2792Pro variant in *BRCA2* (rs28897751) was annotated in ClinVar (VCV000052568.3) as a variant of uncertain significance (VUS). Indeed, at that time, this variant had only been reported in one family [https://www.ncbi.nlm.nih.gov/clinvar/47842750/#evidence]. Most predictions (13 of 15), however, pointed to a clinical significance of the variant (Supplementary Fig. 1C), which was also consistent with the aggressiveness of my PCa, the age of onset (48 years), and my family history. Subsequently, p.Leu2792Pro was evaluated, together with other 138 *BRCA2* VUS, by a functional assay^5^, and found to substantially reduce the homology-directed DNA repair activity of BRCA2, thus having a ≥ 99% probability of pathogenicity. The variant has since (22_Jan_2020) been re-annotated as likely pathogenic in ClinVar, and is present in the OncoKB database (https://www.oncokb.org) as a Level1 (FDA-recognized biomarker predictive of response to an FDA-approved drug) oncogenic mutation for both prostate and ovarian cancers, which can be treated with Olaparib and Rucaparib.

I also analyzed my germline genomic DNA by whole-genome sequencing (WGS), which confirmed the presence of *BRCA2* p.Leu2792Pro and *BRIP1* pArg264Trp heterozygous variants. No additional point mutations or gross rearrangements associated with the risk of aggressive PCa were found.

Search for p.Leu2792Pro in my relatives (2 males and 6 females, aged 31–88) found no carrier, consistent with the fact that no other cancers related to a defect in *BRCA2* were diagnosed in my family except for 3 cases of breast cancer diagnosed later in life (>70 years, 2 individuals were wild-type for the p.Leu2792Pro variant). The only confirmed carrier was me, and, perhaps, my mother. Unfortunately, it was not possible to trace my mother’s tumor specimen to confirm the presence of the mutation in her DNA.

After radical prostatectomy, due to the persistence of a high PSA value (3.95 ng/ml), I started a combined treatment with leuprorelin acetate (3.75 mg 1 intramuscular injection every 28 days) and bicalutamide (50 mg 1 tb po daily for 21 days) to obtain a complete androgen blockade. Less than one week later, a follow-up restaging total body (11 C)Choline-PET/CT scan demonstrated a clear left common iliac proximal adenopathy so I interrupted the treatment with bicalutamide and started a first line of chemotherapy (Docetaxel 75 mg/mq i.v. 1q21 for 6 cycles), maintaining the monthly injection of leuprorelin acetate. This decision was not based on standard guidelines, but the aggressiveness of my cancer, and the fact that it had not been possible to obtain a surgical radicality, suggested that therapy should be as aggressive as possible.

A progressive reduction of my PSA value was seen during the chemotherapy and the good response to the treatment was confirmed by a total body (11C)Choline-PET/CT scan performed after the last cycle. Based on these results, from April to May 2017, I underwent a salvage radiotherapy on the prostate bed and regional pelvic lymph nodes, receiving a total dose of 67.5 Gy and 50 Gy, respectively, delivered in 25 fractions. The treatment was performed with Volumetric Modulated Arc Therapy technique in its RapidArc form, with a simultaneous integrated boost. Prostate bed clinical target volume (CTV_p) was delineated according to EORTC Radiation Oncology Group guidelines^6^.

In July 2018, I suspended the androgen deprivation therapy; in October the PSA was detectable and by March 2019 biochemical recurrence was clearly present (PSA 12.4 ng/ml); another sub-optimal day in my life, although expected. At that stage, the indication was to restart treatment with leuprorelin acetate but just a few months later, when the more sensitive and specific (68)Ga-PSMA-PET/CT became available, I underwent the new imaging that showed bone and peritoneal metastases with minimal peritoneal effusion.

As I was convinced that my *BRCA2* variant was pathogenic (despite the classification as VUS), I was keen to enter a trial protocol with a poly-ADP ribose polymerase (PARP) inhibitor and the oncologists, who were following my case at Humanitas Research Hospital, suggested entering a study at the Istituto Nazionale dei Tumori (INT) in Milan with Olaparib. It was a double-blind phase II trial with abiraterone acetate plus prednisone and olaparib/placebo and I accepted enthusiastically, hoping to be randomly picked up for the active arm of the study. Unfortunately, the protocol did not work for me, and eventually, my disease progressed both biochemically (PSA reached 33.8 ng/ml) and radiologically. Consequently, I left the protocol and discovered that I had been enrolled in the placebo arm. In the meantime, a different clinical trial had opened at the INT (ClinicalTrials.gov Identifier: NCT03840200). In this case I received a combination of an oral AKT inhibitor plus a PARP inhibitor, obtaining, after 9 cycles of treatment, a clear biochemical response (PSA values decreased to about 20-fold compared to baseline, which was >300 ng/ml). In the frame of this study, I was able to have my cancer tissue analyzed by the FoundationOne DX1 test (Foundation Medicine). The results confirmed the presence of the BRCA2 p.Leu2792Pro variant, with no indication on the zygosity; however, the variant was still annotated as VUS, even though its pathogenic role and theranostic relevance had meanwhile been ascertained.

The good biochemical response was confirmed by imaging, since a restaging (68Ga)PSMA-PET/CT after 6 months of treatment documented a reduced extension of viable bone lesions.

In a universe focused on female breast and ovarian hereditary cancers^7^ the importance of identifying male carriers of *BRCA* mutations predisposing to aggressive PCa needs to emerge. Germline deleterious mutations in DDR genes affect up to 16% of metastatic PCa patients^3,8,9^. Above all, *BRCA2* is recognized as the most frequently mutated DDR gene in this setting of patients^3,10^, effecting a more aggressive phenotype and a subsequent poorer outcome^10^.

The ESMO (European Society of Medical Oncology) guidelines recommend referring to NCCN (National Comprehensive Cancer Network) criteria to decide when to perform BRCA testing.^11^ Metastatic PCa at any age represents one of these criteria. However, when it comes to the implementation into clinical practice, the situation varies from country to country. In Italy, according to the current national healthcare system guidelines, men can be screened for *BRCA* germline mutations only if they have been diagnosed with breast cancer or if a pathogenic mutation has already been detected in their family. These directives potentially exclude a not negligible number of PCa patients from the genetic test, who could benefit from an early diagnosis and successful targeted therapies, like PARP inhibitors^12^.

My experience clearly highlights this issue: despite an aggressive PCa at a young age and a first-degree family member affected by breast cancer, our current national guidelines would not have allowed me to be screened for DDR mutations. Such guidelines would have had at least two important implications: (1) No accessibility to testing for DDR mutations and, in case of need, personal screening programmes for other members of my family; (2) my exclusion from clinical trials with PARP inhibitors, with subsequent negative impact on my prognosis.

From my personal experience a few critical points in the clinical management of PCa patients emerge (Fig. 1): (1) an extended family history should always be collected, (2) when a genetic test is performed, a state-of-the-art annotated genetic report should be provided and (3) a medical genetic counseling should be made available. This last point is particularly critical when dealing with germline mutations, to inform patients about the possible implications for their family members and to foster a correct form of family communication, which could increase the uptake of genetic testing by at-risk relatives with important consequences on disease prevention and healthcare costs. In my specific case, I was able to understand the oncogenic potential of my *BRCA2* variant, and all my family members were tested; however, it is easy to understand how inappropriate/incomplete information could impact “standard” patients and general practitioners not specifically trained in medical genetics.

**Fig. 1:**
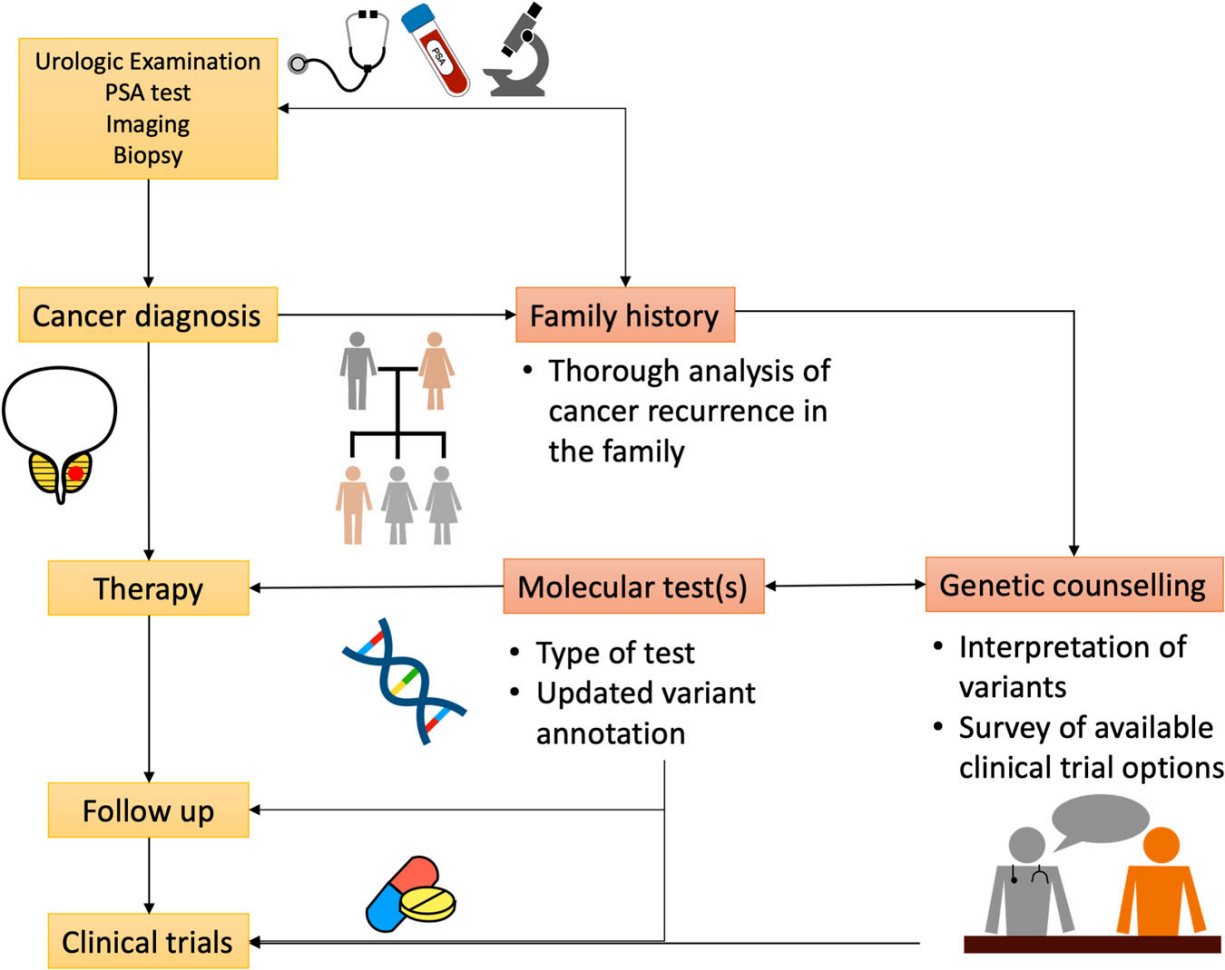
Critical points in the clinical management of PCa patients in the frame of a precision medicine-based approach. The main critical points of attention are highlighted in red.

Understanding the molecular underpinning of my PCa, instead of scaring me, was fundamental in acquiring a proactive attitude towards the disease, which had beneficial effects also on my psychological state. The many decisions that must be taken after a diagnosis of aggressive cancer involve an active participation of the patient that is only possible when clear and complete information has been provided. I was facilitated in this process by my background, but a “lay patient” may become lost in the complex concepts of disease molecular pathogenesis and its implication with therapeutic choices; therefore, a critical point regards the appropriateness of the information that should be given to patients. For this to occur, the interpretation of variants remains a huge problem due to our current imprecise knowledge and the need for new professional figures (genome analyzers) who combine bioinformatics and data management skills with genetic knowledge, essential competences to pre-filter and annotate the variants that must then be communicated by the medical geneticist to the patient.

In conclusion, the ability to translate scientific conceptual breakthroughs into real patient care has to be improved if we are to implement a precision medicine approach towards treating, monitoring, or preventing cancer. Database sharing and common validation procedures are crucial to providing information on actionable mutations, both germline and somatic. A greater effort must be made to convey the medical information to patients in the simplest and most complete way, empowering them to take an active part in therapeutic decisions.

## Methods

The study was approved by the Ethics committee of Istituto Clinico Humanitas. SD signed the appropriate informed consent.

### Whole-exome sequencing

WES library was prepared from 100 ng blood-derived genomic DNA using the Nextera Rapid Capture Exome Enrichment kit v.1.2 (Illumina, San Diego, CA) and sequenced as paired-end 76-bp reads on a NextSeq500 (Illumina). Reads were aligned to hg38 reference genome using the BWA (Burrows-Wheeler Aligner 0.7.17)-MEM program^13^. Single nucleotide variants, (SNVs) identified using GATK (Genome Analysis Toolkit 1.6)^14^, were annotated using OpenCravat v.2.2.1^15^. WES produced >5.6 M paired reads, of which 99.6% aligned to the genome; the 91% of the target region was covered at least 20X (83.2% at least 30X).

### Whole-genome sequencing (WGS)

WGS was performed at Broad Institute, starting from 250 ng DNA on an HiSeq X-Ten (Illumina). The paired 2x150bp reads were aligned against the reference hg38 genome using BWA-MEM, and SNVs identified with GATK. WGS produced 246 M paired reads, of which 99.5% aligned to the genome; the 79.3% of the genome was sequenced at 20X. Germline structural-variant analysis was performed using Lumpy^16^.

## Supplementary information

Supplementary Information

## Data Availability

Considering that the patient described is easily identifiable, the obtained WES and WGS data were not deposited in a public repository. However, upon motivated request, they will be made available.
