# Supplementary material for: How I faced my prostate cancer: a molecular biologist’s perspective
Source: NPJ Precis Oncol. 2021 Sep 24;5:88. doi: 10.1038/s41698-021-00229-5 (PMC8463686; doi:10.1038/s41698-021-00229-5)
Supplement: Supplementary file 1 — Supplementary Information [file 41698_2021_229_MOESM1_ESM.pdf]

a.

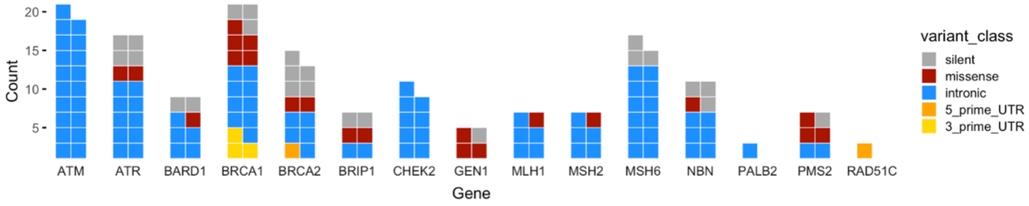

b.

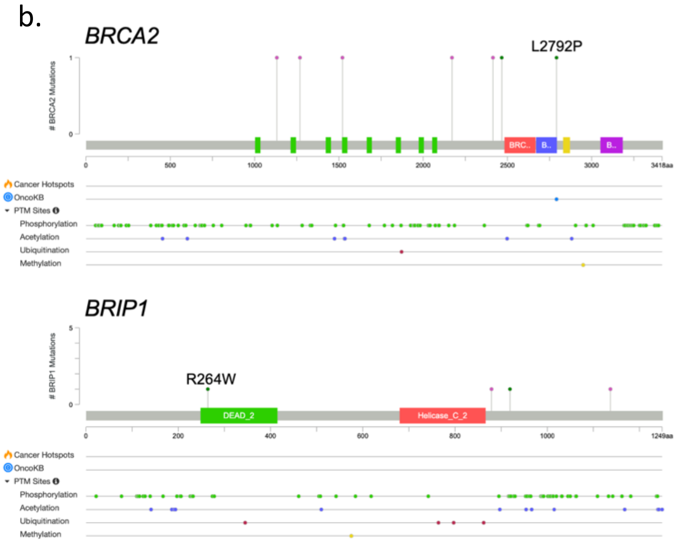

c.

Predictor	*BRIP1* p.R264W Prediction (score)	*BRCA2* p.L2792P Prediction (score)
SIFT	Deleterious (0)	Deleterious (0)
Polyphen	Probably damaging (0.992)	Probably damaging (1)
Mutation Assessor	Medium (0.873)	High (0.961)
Mutation Taster	Polymorphism (0.797)	Disease causing (1)
FATHMM	Tolerated (-3.32)	Damaging (-0.7)
ClinPred	Tolerated (0.031)	Damaging (0.999)
LRT	Neutral (0.001)	Deleterious ($2x10^{-6}$)
MetaLR	Damaging (0.828)	Damaging (0.972)
MetaSVM	Tolerated (-0.003)	Damaging (1.058)
Condel	Deleterious (0.892)	Deleterious (0.945)
PROVEAN	Damaging (-5.79)	Neutral (-2.08)
BayesDel_addAF	Tolerated (0.178)	Damaging (0.955)
BayesDel_noAF	Tolerated (0.621)	Damaging (0.954)
PhD-SNPg	Pathogenic (0.965)	Pathogenic (0.986)
PrimateAI	Tolerated (0.421)	Tolerated (0.646)

Supplementary Figure 1. Genetic variants in DDR genes detected by Whole Exome Sequencing (WES). WES library was prepared from 100 ng blood-derived genomic DNA using the Nextera Rapid Capture Exome Enrichment kit v.1.2 (Illumina, San Diego, CA) and sequenced as paired-end 76-bp reads on a NextSeq500 (Illumina). Reads were aligned to hg38 reference genome using the BWA (Burrows-Wheeler Aligner 0.7.17)-MEM program.[1] Single nucleotide variants, (SNVs) identified using GATK (Genome Analysis Toolkit 1.6),[2] were annotated using OpenCravat v.2.2.1.[3] WES produced >5.6M paired reads, of which 99.6% aligned to the genome; the 91% of the target region was covered at least 20X (83.2% at least 30X). The complete WES data presented in this article are not readily available due to privacy reason, as they contain personally identifiable genetic information. Requests to access the data should be directed to the corresponding author. (**A**) Waffle chart showing the variants detected within DNA repair genes. For each gene, the total number of observed variants and their type is reported. The plot was obtained using R (version

3.6.1), with packages waffle 1.0.1 and ggplot2 3.3.3. (**B**) Lollipop diagrams of *BRCA2* and *BRIP1* showing all detected coding variants (green=missense, purple=silent). The diagram was obtained using the MutationMapper tool on the cBioPortal for Cancer Genomics (chttps://www.cbioportal.org/mutation_mapper). (**C**) Table showing pathogenicity predictions obtained with 15 different software for *BRCA2* p.Leu2792Pro and *BRIP1* p.Arg264Trp.

References:

1. Li H, Durbin R. Fast and accurate short read alignment with Burrows-Wheeler transform. Bioinformatics. 2009;25:1754-60. doi: 10.1093/bioinformatics/btp324.
2. DePristo MA, Banks E, Poplin R, Garimella KV, Maguire JR, Hartl C, Philippakis AA, del Angel G, Rivas MA, Hanna M, McKenna A, Fennell TJ, Kernytsky AM, Sivachenko AY, Cibulskis K, Gabriel SB, Altshuler D, Daly MJ. A framework for variation discovery and genotyping using next-generation DNA sequencing data. Nat Genet. 2011;43:491-8. doi: 10.1038/ng.806.
3. Pagel KA, Kim R, Moad K, Busby B, Zheng L, Tokheim C, Ryan M, Karchin R. Integrated Informatics Analysis of Cancer-Related Variants. JCO Clin Cancer Inform. 2020;4:310-7. doi: 10.1200/CCI.19.00132.
